# Impact of *BRAF^V600E^* mutation on aggressiveness and outcomes in adult clonal histiocytosis

**DOI:** 10.3389/fimmu.2023.1260193

**Published:** 2023-09-22

**Authors:** Jerome Razanamahery, Amelie Godot, Vanessa Leguy-Seguin, M. Samson, Sylvain Audia, Bernard Bonnotte

**Affiliations:** ^1^ Department of Internal Medicine and Clinical Immunology, Dijon University Hospital, Dijon, France; ^2^ Department of Internal Medicine, Besancon University Hospital, Besancon, France

**Keywords:** histiocytosis, Erdheim-Chester disease, Langerhans cell histiocytosis (LCH), Rosai Dorfman disease, BRAF

## Abstract

Histiocytoses encompass a wide spectrum of diseases, all characterized by tissue infiltration by CD68+ histiocytes. Most adult histiocytoses are considered clonal diseases because they highlight recurrent somatic mutations in the MAP-kinase pathway gene, primarily *BRAF*. The presence of *BRAF* mutation is associated with widespread disease in children with Langerhans cell histiocytosis (LCH) or cardiovascular/neurological involvement in Erdheim–Chester disease (ECD). Nevertheless, few data are available on adult clonal histiocytosis. This is why we have conducted a retrospective study of all patients with clonal histiocytosis in our institution and present the data according to the presence of *BRAF* mutation. Among 27 adult patients (10 ECD, 10 LCH, 5 Rosai–Dorfman disease (RDD), and 3 mixed ECD/LCH), 11 (39%) have *BRAF* mutation with gain of function (n = 9) and deletion (n = 2). Those patients had frequent multicentric disease with risk organ involvement, especially the brain and cardiovascular system. They had frequent associated myeloid neoplasms (mostly chronic myelomonocytic leukemia) and received more frequently targeted therapy as the front-line therapy. Nevertheless, its presence did not affect the overall survival or relapse-free survival probably due to the emergence of efficient therapies. To conclude, rapid and accurate molecular establishment in adult clonal histiocytoses is crucial because *BRAF^V600E^* mutation correlates with multicentric disease with organ involvement and incomplete metabolic response.

## Introduction

Histiocytoses are clonal hematopoietic disorders characterized by CD68+ histiocyte tissue infiltration causing a wide clinical spectrum of diseases (1). The recent identification of somatic mutations in the Mitogen-Activated Protein Kinase (MAP-kinase) pathway gene, especially *BRAF^V600E^* in most tissue samples from adult histiocytoses [Langerhans cell histiocytosis (LCH) (2), Erdheim–Chester disease (ECD) (3), and Rosai–Dorfman disease (RDD) (4)], led to consider these diseases as myeloid neoplasm (5). Interestingly the genetic landscape analysis of various cancers, such as melanoma, colon cancer, lung cancer, and more recently histiocytic neoplasms, has highlighted the pivotal role of *BRAF^V600E^* gene, which is considered an important cornerstone in the development of human cancer. Its presence is associated with disseminated disease, neurodegeneration, and resistance to front-line therapy in pediatric LCH patients (6, 7). In adults, LCH is mainly restricted to the lung, making *BRAF^V600E^* testing difficult due to the risk of a pulmonary biopsy procedure and a low DNA quantity when available. Hence, few data are available regarding the impact of *BRAF^V600E^* mutation in adult extrapulmonary LCH. *BRAF^V600E^* mutation has also been described in ECD, a rare adult non-Langerhans cell histiocytosis. Its presence is significant because patients with cardiac and neurological locations are frequently *BRAF*-mutated (8). Because LCH, ECD, and RDD are characterized by clonal origins, we aimed to investigate the impact of *BRAF^V600E^* mutation on aggressiveness and outcomes in a cohort of adult histiocytoses. This study describes the distinct characteristics and outcomes of various adult histiocytoses separately, followed by an analysis according to *BRAF^V600E^* mutational status.

## Method

This is a single-center retrospective study (local ethics board approved) for which informed consent was waived. Patients included were at least 18 years old with biopsy-confirmed histiocytosis diagnosed from 1990 to 2022. Tissue infiltration by histiocytes (CD68+) and hematoxylin and eosin staining for CD207, CD1a, and S100 analyses were mandatory. The diagnosis of histiocytoses was performed according to expert guidelines (9–11). LCH patients had compatible clinical manifestations (i.e., skin, liver, lung, colon, endocrine, or brain) and tissue biopsy showing infiltration by CD68+, CD1a+, and CD207+ histiocytes. Biopsy was mandatory except for isolated pulmonary LCH after exclusion for differential diagnosis. ECD patients had iconic features (long bone osteosclerosis, peri-renal infiltration, and vascular sheathing) along with histology showing CD68+ and CD1a− histocytes accompanied by varying degrees of fibrosis. RDD patients had typical histology features with CD68+ and CD1a− with emperipolesis.

*BRAF^V600E^* status was established using droplet PCR (12) and next-generation sequencing (NGS) technology if the DNA quantity was sufficient (13). The liver, spleen, and bone marrow were risk organs for LCH patients, and the cardiovascular system was at risk for ECD patients. The brain was a risk organ for all patients.

The treatment approach was based on symptoms and organ involvement according to guidelines and expert consensus. For patients with multisystemic Langerhans cell histiocytosis (LCH), chemotherapy was the first-line therapy, while immunosuppressive agents were considered for cases of isolated skin LCH. All patients with LCH were advised to stop smoking.

For the ECD patients, treatment options included interferon, biologic agents (such as IL-1 or TNF-alpha inhibitors), or targeted therapy for severe cases (i.e., cardiovascular or neurological involvement). Patients with RDD received treatment when presenting symptomatic lymphadenopathy or autoimmune-associated conditions. Non-symptomatic patients without risk of organ infiltration were closely monitored. For all patients, targeted therapies were proposed following relapse or failure of conventional treatments. Specifically, patients with the *BRAF^V600E^* mutation received a BRAF inhibitor (vemurafenib) at a dose of 420 mg twice daily, while those without the mutation received a MEK inhibitor (cobimetinib) at a dose of 400 mg for 21 days within a 28-day cycle. All treatment decisions were validated with the reference center for histiocytosis at Pitié-Salpétrière Hospital.

Disease activity was assessed using ^18^fluorodeoxyglucose positron tomography (according to Positron Emission Tomography Response Criteria In Solid Tumors (PERCIST) criteria). Complete metabolic response was defined by the normalization of all lesions to at or below the standardized uptake value (SUV) of the liver background. Partial metabolic response was defined by a ≥50% decrease from the baseline sum of all target lesions’ SUV. Progressive metabolic disease was defined by a ≥50% increase in the nadir sum of all target or new evaluable lesions’ SUV. Stable metabolic disease was defined if the patient did not meet the previous criteria. To evaluate the efficacy response following front-line therapy, patients with either a complete metabolic response or a partial metabolic response were classified as responders. In contrast, patients with stable or progressive metabolic disease were categorized as non-responders. The complete diagnosis procedures are reported in **Supplemental Data**. Comparison between groups was performed using the Mann–Whitney test, chi2, or Fisher’s test. For multiple comparison groups, an analysis of variance (ANOVA) test with the Kruskal–Wallis procedure was used followed by Dunn’s multiple comparison. Multiple logistic regression analysis for factors associated with *BRAF* mutation was performed with all variables with a p-value <0.2 in simple logistic regression. Statistical significance was set at p < 0.05 (two-sided). Statistical analyses were performed using GraphPad Prism software V.10 (GraphPad, San Diego, CA, USA).

## Results

Twenty-eight adult patients were included in the study. Ten patients had ECD, 10 LCH, 5 RDD, and 3 mixed “ECD/LCH”. Comparison between the four groups showed differences in terms of phenotype and disease course. ECD patients had a more frequent retroperitoneal involvement than LCH patients (80% vs. 0%; p = 0.024) and a more frequent digestive localization than RDD patients (70% vs. 0%; p = 0.046). Their mesentery biopsies revealed histiocyte infiltration more frequently than in LCH patients (50% vs. 0%; p = 0.0249). However, ECD patients had limited cutaneous histiocytic infiltration as compared with mixed “ECD/LCH” (10% vs. 100%; p = 0.024). Patients with LCH had more frequent endocrine system involvement (“arginine vasopressin deficiency”) than ECD patients (60% vs. 10%; p = 0.048). In addition, RDD patients had more lymph node involvement than ECD (40% vs. 0%; p = 0.03) and LCH patients (40% vs. 0%; p = 0.03) as confirmed by histological samples. They also had a lower rate of risk organ involvement compared to “mixed ECD/LCH” patients (0% vs. 100%; p = 0.042). At the last follow-up, RDD patients more often achieved a complete metabolic response compared with ECD patients (100% vs. 10%; p = 0.0050), while ECD patients often had more stable metabolic disease compared with LCH patients (60% vs. 0%; p = 0.03). Regarding the mutational landscape, 11 patients (39%) had *BRAF^V600E^* mutation (5 LCH, 4 ECD, and 2 “ECD/LCH”) with gain-of-function mutation on exon 15 (n = 9) and deletion in exon 12 (n = 2). *BRAF-*mutated patients also had mutations in *KRAS*, *TET2*, *DNMT3A*, *ASXL1*, and *SRSF2* within biopsy tissue. *BRAF* wild-type patients had *MAP2K1* gene mutation in exon 2 [RDD (n = 2) and ECD (n = 1)] (**Table 1**, **Supplementary Table 1**). There is no statically relevant correlation between the frequency of BRAF^V600E^ mutation and the type of histiocytosis. All patients had overexpression of phosphorylated extracellular signal-regulated kinase (phospho-Erk) in tissue samples. One *BRAF* wild-type patient had overexpression of both the “Colony stimulating factor 1 receptor” (*CSF1R*) and “Programmed death ligand 1” (*PDL1*). Three patients could not undergo NGS analysis due to insufficient DNA quantity.

**Table 1:** Characteristics of patients with histiocytoses according to *BRAF^V600E^* status.

	Overall cohort (n = 28)	*BRAF^V600E^* (n = 11)	*BRAF^wt^* (n = 17)	p-Value
Age (median IQR)	62 [39–71]	68 [60–72]	58 [34–68]	0.1014
Type of histiocytosis				
ECD	10 (36%)	4 (37%)	6 (35%)	>0.999
LCH	10 (36%)	5 (45%)	5 (29%)	0.443
Mixed (ECD/LCH)	3 (11%)	2 (18%)	1 (6%)	0.5433
RDD	5 (17%)	0 (0%)	5 (30%)	0.1247
Presentation				
Unicentric	10 (35%)	1 (9%)	9 (52%)	0.0407^*^
Multicentric	18 (65%)	10 (91%)	8 (48%)	0.0407^*^
Risk organ involvement	13 (46%)	9 (81%)	4 (23%)	0.0056^**^
Location of histiocytosis				
Bone	19 (67%)	9 (81%)	12 (70%)	0.6683
Cardiovascular	6 (21%)	5 (45%)	1 (5.8%)	0.0221^*^
Pulmonary	5 (17%)	2 (18%)	3 (17.6%)	>0.9999
Endocrine	8 (28%)	6 (54%)	2 (11.7%)	0.0299*
Digestive	9 (32%)	5 (45%)	4 (23%)	0.4087
Retroperitoneal	10 (35%)	5 (45%)	5 (29.47%)	0.4443
Lymph node	3 (11%)	0 (0%)	3 (17.6%)	0.2579
Eye/orbital	6 (21%)	4 (36%)	2 (11.7%)	0.1741
Neurological	6 (21%)	5 (45%)	1 (5.8%)	0.0221*
Hematological conditions				
Myeloproliferative disorders	4 (14%)	4 (36%)	0 (0%)	0.0462*
Clonal hematopoiesis	9 (32%)	3 (27%)	6 (35%)	0.1789
Treatment				
Watchful waiting		0 (0%)	5 (29%)	0.1247
Front line therapy		11 (100%)	12 (71%)	0.1247
Front line therapy				
Conventional therapies (Peg-Inf, steroids, MTX, and chemotherapy)	13 (46%)	6 (54%)	7 (41%)	0.700
Biological agents	3 (10%)	0 (0%)	3 (17%)	0.2579
Targeted therapy	5 (17%)	4 (36%)	1 (5.8%)	0.0618
Metabolic response at last follow-up				
Complete metabolic response	8 (28%)	0 (0%)	8 (47%)	0.0077**
Partial metabolic response	5 (17%)	4 (36%)	1 (5.8%)	0.1206
Stable metabolic disease	8 (28%)	5 (45%)	3 (17%)	0.1936
Progressive metabolic disease	5 (17%)	2 (4%)	3 (17%)	>0.999
Death at last follow-up	5 (17%)	4 (36%)	1 (5.8%)	0.0618

ECD, Erdheim–Chester disease; LCH, Langerhans cell histiocytosis; RDD, Rosai–Dorfman disease; IQR, interquartile range.

*, P<0.05; **, P<0.005.

*BRAF^V600E^*-mutated patients had multicentric disease (91% vs. 48%; p = 0.041) with “risk organ” involvement (81% vs. 23%; p = 0.005), particularly the cardiovascular system (45% vs. 5.8%; p = 0.022) and the brain (45% vs. 5.8%; p = 0.022). They also had frequent endocrine involvement (54% vs. 11%; p = 0.029). Biological tests at diagnosis were similar (data not shown).

The occurrence of “risk organ” involvement remained higher in *BRAF^V600E^*-mutated patients after the exclusion of RDD patients (81% vs. 33%; p = 0.036). However, there was only a trend toward cardiovascular and brain involvement (45% vs. 8%; p = 0.06 for both). The *BRAF^V600E^*-mutated patients also had myeloproliferative neoplasm (three chronic myelomonocytic leukemia and one JAK2-mutated essential thrombocythemia) compared to the *BRAF* wild-type group (36% vs. 0%; p = 0.046). The frequency of clonal hematopoiesis (characterized by the presence of myeloid gene mutation on bone marrow without morphologic evidence for myelodysplastic or myeloproliferative disease) was similar in both groups. Patients in the study received several lines of treatment ranging from none to six depending on relapse rate. All *BRAF*-mutated patients had symptoms requiring treatment ranging from non-steroidal inflammatory agents (n = 1) for LCH and from conventional therapies (n = 6) to targeted therapies (n = 4) for severe cases. Some *BRAF* wild-type patients received no treatment, such as those with non-symptomatic RDD (n = 4), while others received surgery (n = 1), conventional agents including chemotherapy (n = 7), or biological agents (n = 3). Among *BRAF^V600E^* patients receiving first-line targeted therapy were multisystemic ECD (n = 3), LCH (n = 1), and mixed ECD/LCH (n = 1) with risk organ involvement. One BRAF^wt^ patient received first-line treatment with cobimetinib due to contraindications to other ECD therapies. Hence, the use of targeted therapy as first-line treatment tends to be more frequent in *BRAF^V600E^*-mutated patients (36% vs. 6%; p = 0.062). Efficacy of first-line therapy was similar between groups (55% vs. 82%; p = 0.1998). Some patients received targeted therapies during the disease course [ECD (n = 4) and LCH (n = 1)] after a relapse. Almost all patients (90%) treated with targeted therapies had a partial metabolic response after treatment initiation, but no patient achieved a complete metabolic response. Furthermore, the use of BRAF inhibitor was responsible for CMML worsening in three patients requiring treatment discontinuation but no specific treatment such as azacytidine or hydroxycarbamide. Subsequent administration of a MEK inhibitor successfully restored the metabolic response associated with the normalization of the monocyte count.

The median [IQR] follow-up was 113 [78–180] months. *BRAF* wild-type patients had a more complete metabolic response (48% vs. 0%; p = 0.007), but the relapse rate was not influenced by *BRAF^V600E^* status. Death tends to be more frequent in *BRAF* patients (36% vs. 5.8%; p = 0.061). The cause of death was infections, except for two patients (one in both groups) who died from disease progression. The rate of complete metabolic response in *BRAF* wild-type patients remained higher after the exclusion of RDD patients (41% vs. 0%; p = 0.004).

**Figure 1:**
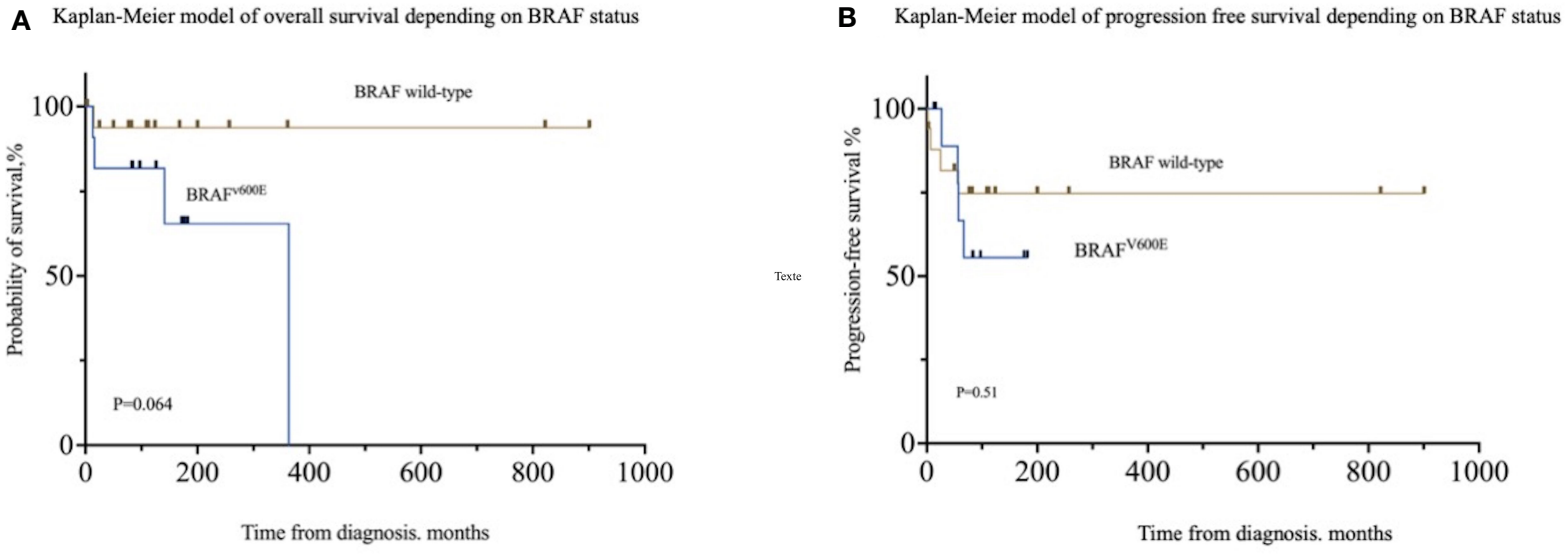
Patient outcomes according to *BRAF^V600E^* status. **(A)** Overall survival in patients depending on *BRAF^V600E^* status. **(B)** Progression-free survival depending on *BRAF^V600E^* status.

Univariate logistic regression analysis showed that *BRAF^V600E^* was associated with “risk-organ” involvement (OR = 14.63; 95% CI [2.555–128]; p = 0.005), especially brain involvement (OR = 12.50; 95% CI [1.589–268]; p = 0.035), multicentric disease (OR = 11.25; 95% CI [1.610–230.7]; p = 0.036), and endocrine involvement (OR = 8.40; 95% CI [1.422–72.31]; p = 0.028) but not in multivariate analysis (**Supplementary Table 2**). Neither overall survival (HR: 5.574; 95% CI [0.90–33.5]; p = 0.064) nor progression-free survival (HR: 1.613; 95% CI [0.3847–6.760]; p = 0.51) was impacted by *BRAF^V600E^* status (**Figure 1**).

## Discussion

In this study, we present the distinctive features of 28 adults with various forms of clonal histiocytosis, combined with an exploration of the impact of the presence of *BRAF^V600E^* mutation on presentation and outcome.

In the first part of the analysis, we observed that all cases of clonal histiocytosis exhibited a unique phenotype consistent with the literature (1). Patients diagnosed with Rosai–Dorfman disease had mutations in genes related to the MAP-kinase pathway, excluding BRAF^v600E^. Notably, their disease course appeared distinct compared to “L-group histiocytosis”. Our data showed that *BRAF^V600E^* mutation was associated with multicentric disease and risk organ involvement. This phenotype is in line with pediatric LCH (6) (7) studies and ECD cohort (8). Excluding RDD patients, the presence of *BRAF^V600E^* correlated with risk organ involvement irrespective of the type of “L-group histiocytosis”.

Our low frequency of *BRAF^V600E^* mutation can be attributed to the presence of RDD patients, in which *BRAF^V600E^* mutation has only been reported in two cases. (14). The mutational landscape regarding *BRAF^V600E^* and other MAP-kinase pathway genes is similar to the literature. In addition, the expression of phospho-Erk in all patients confirms the activation of the MAP-kinase pathway cascade (15) and confirms the clonal origins of those histiocytoses. In this series, the NGS analysis identified additional mutations of clonal hematopoiesis genes on biopsy samples, confirming a continuum between myeloid hematopoiesis and histiocytoses, especially in *BRAF-*mutated patients (1). This finding is reinforced by the occurrence of authentic myeloid neoplasms in *BRAF*-mutated patients and the efficacy of targeted therapy on both diseases in these patients. The recent advances in ontogeny understanding of histiocytic disorders partly explain the clinical presentation. In LCH, *BRAF^V600E^* mutation in bone marrow progenitors gives rise to mutated clones infiltrating myeloid dendritic cells, which infiltrate virtually all tissues (16). The same mechanism is proposed in ECD with a predominant involvement of CD14+ monocytes (17) rather than dendritic cells and also an abnormal activation of “trained immunity” mediated by *BRAF* (18). The understanding of the brain involvement mechanism has yet to be elucidated. It may result from a continuum in the blood–brain barrier between *BRAF^V600E^* monocytes deriving from yolk sack (19) progenitors and bone marrow (20).

In terms of outcome, the complete metabolic response achievement is influenced by the absence of the *BRAF^V600E^* mutation. Nevertheless, it has no impact on overall survival probably due to the efficacy of the drugs. Hence, targeted therapies have revolutionized the prognosis of the disease (21–24). In our cohort, these treatments were efficient in almost all patients, although they can cause various side effects, prompting consideration for a transition from BRAF inhibitors to MEK inhibitors in order to regain efficacy. These treatments may be of interest as a front-line therapy to avoid several lines of chemotherapy regimens in elderly patients who die mainly from infections. Furthermore, these treatments reduce the mutational *BRAF^V600E^* load and thus the risk of brain invasion by mutated clones causing delayed neurodegeneration. However, it is important to notice that these treatments are only suspensive and not curative for these diseases. The absence of complete metabolic disease in this cohort confirms this postulate and is in line with the literature (22). Other signaling pathways for which we have specific inhibitors (AKT/mTOR, *ALK*, and *CSF1R*) (25, 26) now pave the way for targeted therapies rather than conventional chemotherapy regimens in adult clonal histiocytosis.

This study has certain limitations. The retrospective nature and the data collection bias the analysis. ^18^FDG-PET or MRI was not available for the patients with the earliest diagnosis. The choice of therapeutic approach was influenced by the recent upgrade of targeted therapies, which were only considered as salvage therapy previously.

Nevertheless, the main strength of this study is the thorough molecular (including bone marrow) evaluation in most adult clonal histiocytoses mixing adult extrapulmonary LCH, ECD, and RDD, followed by a morphologic and metabolic response to assess the impact of *BRAF^V600E^* mutation.

## Conclusion

Rapid and accurate molecular establishment in adult clonal histiocytoses is crucial because *BRAF^V600E^* mutation correlates with multicentric disease with organ involvement and incomplete metabolic response.

## Data availability statement

The original contributions presented in the study are included in the article/**Supplementary Materials**, further inquiries can be directed to the corresponding author.

## Ethics statement

The studies involving humans were approved by Dijon university hospital local ethics committe. The studies were conducted in accordance with the local legislation and institutional requirements. The participants provided their written informed consent to participate in this study.

## Author contributions

JR: Conceptualization, Data curation, Formal Analysis, Investigation, Methodology, Validation, Writing – original draft, Writing – review & editing. AG: Data curation, Writing – review & editing. VL-S: Data curation, Writing – review & editing. MS: Data curation, Writing – review & editing. SA: Data curation, Writing – review & editing. BB: Conceptualization, Formal Analysis, Investigation, Methodology, Supervision, Writing – review & editing.
