# Supplementary material for: Impact of BRAFV600E mutation on aggressiveness and outcomes in adult clonal histiocytosis
Source: Front Immunol. 2023 Sep 22;14:1260193. doi: 10.3389/fimmu.2023.1260193 (PMC10556468; doi:10.3389/fimmu.2023.1260193)
Supplement: Supplementary file 1 [file DataSheet_1.docx]

**Diagnosis procedure for histiocytoses**

Erdheim-Chester disease (ECD) patients had iconic features (long bones involvement and/or perinephric fat infiltration and/or vascular sheathing of the adventitia of vessels) with compatible histology (CD68^+^, CD1a^-^ histiocytes). Tissue biopsy presented gain of function mutation in genes of the MAP-kinase pathway (if available) or at least moderate to strong expression of phosphorylated-Erk.^1^ Rosai-Dorfman Disease (RDD) patients had typical histology with tissue biopsy disclosing infiltration by CD68^+^, S100^+^, CD1a^-^ histiocytes with enlarged nuclei and lesions of emperipolesis^2^. Langerhans Cell Histiocytosis (LCH) patients had biopsy showing an inflammatory infiltrate associated with CD68^+^, CD1a+, S100^+^ histiocytes or typical pulmonary images (after excluding differential diagnosis) and compatible broncho-alveolar lavage in the absence of tissue biopsy^3^.

Professor Jean-François Emile (Ambroise-Paré Hospital) centrally reviewed tissue biopsies for NGS technology when possible and Professor Julien Haroche and Fleur Cohen-Aubart (Pitié-Salpétrière) provide clinical expertise for uncertain case.

Histology was performed on 4-micrometer thick tissue sections after staining with Hematoxylin & Eosin and immunohistochemistry, including at least CD1a, S100, and CD68, CD163 primary antibodies. Detection of mutations was performed on tissue biopsies infiltrated by histiocytosis. Tumor DNA was extracted from formalin-fixed and paraffin-embedded tissues. Detection of *BRAF^V600E^* mutation was performed as previously described using picodroplet digital PCR^4,5^. Detection of other mutations was performed using targeted next-generation sequencing (NGS). Samples were analyzed using MiSeq (Illumina^®^) after preparing the Custom Amplicon Low Input Kit libraries. The targeting genes are listed as follows: *AKT1, ALK, ARAF, ASXL1, BRAF, CALR, CBL, CDK4, CDKN1B, CDKN2A, CEBPA, CSF3R, CTNNB1, DNMT3A, EGFR, EZH2, FLT3, GATA2, GNA11, GNAQ, GNAS, HERC1, HRAS, IDH1, IDH2, JAK2, JAK3, KIT, KRAS, KTM2D, MAML3, MAMLD1, MAP2K1, MAP2K2, MAP2K3, MAP2K4, MAP2K6, MAP3K1, MAP3K8, MAP3K9, MAP3K10, MAP3K19, MAP4K4, MAPK1, MAPK11, MAPK9, MPL, NF1, NOTCH1, NOTCH2, NPM1, NRAS, PDGFRA, PIK3CA, PP6C, PTEN, PTPN11, RAC1, RAF1, RIT1, RUNX1, SETBP1, SRSF2, STAG2, STK19, SYNGAP1, TAOK1, TAOK2, TET2, TP53, U2AF1, WT1, ZRSR2.*

**Disease activity staging of histiocytoses**

Disease activity was established using the last metabolic evaluation with ^18^Fluorodeoxyglucose positron emission tomography-computed tomography (^18^FDG-PET-CT) according to PERCIST criteria^6,7,8^. Complete metabolic response is defined by normalization of all lesions to at or below Standardized Uptake Value (SUV) of liver background_._ Partial metabolic response is defined by a ≥50% decrease from baseline sum of all target lesions SUV. Progressive metabolic disease is defined by a ≥50% increase in nadir sum of all target or new evaluable lesions SUV. Stable metabolic disease is defined if patient did not meet previous criteria. Patients were separated depending on metabolic response as "complete metabolic response", "partial metabolic response", "stable metabolic disease” or "progressive metabolic disease”. We have considered patients with "complete metabolic response" and "partial metabolic response" as responders and patients with "stable metabolic disease" and "progressive metabolic disease" as non-responders for the analysis.

**Determination of clonal hematopoiesis:**

The myeloid gene panel analyzed by next generation sequencing (NGS) included: *ASXL1, BCOR, BCORL1, CALR, CBL, CSF3R, DNMT3A, ETV6, EZH2, FLT3, GATA2, IDH1, IDH2, JAK2, KIT, KRAS, MPL, NIPBL1, NPM1, NRAS, PHF6, PTPN11, RAD21, RIT1, RUNX1, SETBP1, SF3B1, SMC1A, SMC3, SRSF2, STAG2, TET2, TP53, U2AF1, WT1, ZRSR2*. Variant allele frequency (VAF) was considered significant over a threshold of 2%. The presence of at least one myeloid gene mutation in bone marrow analysis without morphological evidence of hematological neoplasm defined clonal hematopoiesis (CH).^9^

**Statistical analysis**

Groups were compared by using Fisher’s exact test for the qualitative data and the Student *t*-test or Mann–Whitney test for the continuous data for univariate analysis.

For multiple comparison group, we used an analysis of variance (ANOVA) test with Kruskal-Wallis procedure followed by Dunn’s multiple comparison. All reported *p*-values were two-sided, and a *p*-value <0.05 was considered statistically significant.

We have performed a logistic regression analysis to identify factor associated with *BRAF^V600E^* status in univariate analysis. All factors with P.value <0.2 in univariate analysis were analyzed in multivariate analysis. For all graph analysis, significant *p*-value are presented as follows: *p<0.05; **p<0.001.

Statistical analysis was performed using GraphPad software, V.10 (GraphPad, San Diego, California, USA).

**References**:

1. Goyal G, Heaney ML, Collin M, et al. Erdheim-Chester disease: Consensus recommendations for the evaluation, diagnosis, and treatment in the molecular era. *Blood*. 2020;

2. Abla O, Jacobsen E, Picarsic J, et al. Consensus recommendations for the diagnosis and clinical management of Rosai-Dorfman-Destombes disease. *Blood*. 2018;131(26):2877–2890.

3. Rodriguez-Galindo C, Allen CE. Langerhans Cell histiocytosis. *Blood*. 2020;

4. Diamond EL, Durham BH, Haroche J, et al. Diverse and Targetable Kinase Alterations Drive Histiocytic Neoplasms. *Cancer Discov*. 2016;6(2):154–165.

5. Melloul S, Hélias-Rodzewicz Z, Cohen-Aubart F, et al. Highly sensitive methods are required to detect mutations in histiocytoses. *Haematologica*. 2019;104(3):e97–e99.

6. Tirkes T, Hollar MA, Tann M, et al. Response criteria in oncologic imaging: review of traditional and new criteria. *Radiographics*. 2013;33(5):1323–1341.

7. Diamond EL, Subbiah V, Lockhart AC, et al. Vemurafenib for BRAF V600-Mutant Erdheim-Chester Disease and Langerhans Cell Histiocytosis: Analysis of Data From the Histology-Independent, Phase 2, Open-label VE-BASKET Study. *JAMA Oncol*. 2018;4(3):384–388.

8. Diamond EL, Durham BH, Ulaner GA, et al. Efficacy of MEK inhibition in patients with histiocytic neoplasms. *Nature*. 2019;567(7749):521–524.

9. Steensma DP, Bejar R, Jaiswal S, et al. Clonal hematopoiesis of indeterminate potential and its distinction from myelodysplastic syndromes. *Blood*. 2015;126(1):9–16.
