# Supplementary material for: Impact of BRAFV600E mutation on aggressiveness and outcomes in adult clonal histiocytosis
Source: Front Immunol. 2023 Sep 22;14:1260193. doi: 10.3389/fimmu.2023.1260193 (PMC10556468; doi:10.3389/fimmu.2023.1260193)
Supplement: Supplementary file 2 [file Table_1.docx]

**Supplementary table 1:** Characteristic of the patients with molecular analysis

| **case** | **Age at diagnosis** | **sex** | **Type of histiocytosis** | **Localization of histiocytosis** | **Mutational status on tissue infiltrated by histiocytes** | **Myeloid neoplasm** | **Clonal hematopoiesis** | **Bone marrow mutation** | **treatment** | **Disease activity** | **Alive at last-follow up** |
| --- | --- | --- | --- | --- | --- | --- | --- | --- | --- | --- | --- |
| 1 | 71 | M | ECD | mesentery, bone, CNS, peri-renal, heart | *BRAF c.1799T>A,p.(Val600Glu),*  *KRAS c.437C>T , p(Ala146 Val)*  *TET2 c.3622A>T, p.(Lys1208*)* | CMML | 0 | *TET2/ZRS2/KRAS/NRAS/BRAF* | Vemurafenib  Kineret  cobimetinib | SMD | yes |
| 2 | 68 | F | ECD | mesentery, bone, CNS, peri-renal, heart, bone marrow | *BRAF c.1799T>A,p.(Val600Glu),* | CMML | 0 | *TET2/SRSF2/CLB/NRAS* | Vemurafenib  Cobimetinib | SMD | yes |
| 3 | 71 | M | ECD | heart, bones, vessels, mesentery, peri-renal | *BRAF c.1799T>A,p.(Val600Glu),* | ET (JAK2+) | 0 | *JAK2/TET2/NF1* | Interferon  Cobimetinib | SMD | yes |
| 4 | 61 | F | LCH | liver, endocrine, bone, skin | *BRAF c.1457_1471del,p.(486_490del)*  *DNMT3 A c.1742G>C,p.(Trp581Ser)* | 0 | 1 | *TET2* | Vinblastine/steroid  Cobimetinib | PMR | yes |
| 5 | 72 | F | LCH/ECD | bone, lung , skin, vessels | *BRAF c.1799T>A,p.(Val600Glu),* | 0 | 1 | *KRAS/SH2B3/SRSF2/TET2* | vemurafenib | SMD | no |
| 6 | 81 | F | ECD | bone, heart, vessel, CNS, peri-renal | *BRAF c.1799T>A,p.(Val600Glu),* | 0 | 1 | *ASXL1/NF1/TET2/U2AF1* | Interferon  vemurafenib | PMR | no |
| 7 | 59 | M | LCH | bone, lung, pituitary gland | *BRAF c.1799T>A,p.(Val600Glu),* | 0 | 0 | *0* | Steroids/vinblastin  Cladribine | PMR | yes |
| 8 | 78 | F | LCH | colon, skin, pituitary gland, liver, bone marrow | *BRAF c.1799T>A,p.(Val600Glu),* | CMML and AML | 0 | *TET2* | vemurafenib | NA | no |
| 9 | 60 | F | LCH/ECD | pituitary gland, skin, bone,heart,aorta,peri-renal,brain, optic nerve | *BRAF c.1799T>A,p.(Val600Glu),* | 0 | 0 | *0* | Steroid  Vinblastin  Methotrexate  Interferon  Rituximab  Vemurafenib | SMD | no |
| 10 | 29 | F | LCH | pituitary gland, eye, brain | *BRAF c.1457_1471del,p.(486_490del)* | 0 | 0 | *0* | cladribin | PMD | yes |
| 11 | 64 | F | LCH | bone | *BRAF c.1799T>A,p.(Val600Glu),* | 0 | 0 | *0* | NSAID | PMD | yes |
| 12 | 69 | M | RDD | perirenal | *MAP2K1 c.395C>T, p(Ala132Val)* | 0 | 1 | *TET2/ASXL1/DNMT3A/JAK2* | Rituximab | CMR | yes |
| 13 | 39 | M | RDD | skin,bone,eyes, vessels | *MAP2K1 c.361T>A* | 0 | 0 | *0* | steroids | CMR | yes |
| 14 | 73 | M | ECD | mesentery, peri-renal | *MAP2K1 c.209A>G, p(Lys97Arg)* | 0 | 0 | *0* | none | SMD | yes |
| 15 | 80 | M | LCH | skin | *TET2 c.270C>T , p.(Gln904Ter)*  *ASXL1 c.1773C>A,p.(Tyr591Ter)*  *SRSF2 c.284C>A, p.(Pro95His)* | 0 | 1 | *ASXL1/TET2/SRSF2* | Methotrexate | SMD | yes |
| 16 | 24 | F | ECD | vessels, bone, sinus | No mutation | 0 | 0 | *0* | Kineret  Cobimetinib  Steroid/ciclosporin/MMF after transplantation | PMD | no |
| 17 | 67 | M | RDD | bone | No mutation | 0 | 0 | *0* | Steroids | CMR | yes |
| 18 | 12 | M | LCH | bone, skin, endocrine, lung | No mutation | 0 | 0 | *0* | Steroid/vinblastin | PMD | yes |
| 19 | 64 | M | ECD | bone, peri-renal, mesentery | No mutation | 0 | 1 | *TET2* | Cobimetinib | SMD | yes |
| 20 | 58 | F | ECD | bone, mesentery, peri-renal | No mutation | 0 | 1 | *DNMT3A* | Interferon  Kineret  Infliximab  Cobimetinib | PMD | yes |
| 21 | 67 | M | LCH | lung | No mutation | 0 | 0 | *0* | none | CMR | yes |
| 22 | 18 | F | LCH | lung, hypophysis, bones | No mutation | 0 | 0 | *0* | none | CMR | yes |
| 23 | 62 | F | RDD | skin, lymph node | No mutation | 0 | 0 | *0* | none | CMR | yes |
| 24 | 79 | F | RDD | lymph node | No mutation | 0 | 0 | *0* | none | NA | yes |
| 25 | 54 | M | ECD | bone, skin | No mutation | 0 | 1 | *JAK2* | interferon | CMR | yes |
| 26 | 37 | M | LCH/ECD | bone, skin, brain | No mutation | 0 | 1 | *U2AF1* | Vinblastin/steroid  Cladribin | CMR | yes |
| 27 | 58 | F | ECD | Bone, skin, vascular, peri-renal, | No mutation | 0 | 0 | 0 | Infliximab  Kineret | SMD | yes |
| 28 | 31 | F | LCH | brain | No mutation | 0 | 0 | 0 | Surgical resection | CMR | yes |

ECD : Erdheim Chester Disease, LCH : Langerhans cell histiocytosis, RDD: Rosai-Dorfman Disease, CMML: chronic myelomonocytic leukemia, ET: essential thrombocythemia. Disease activity was established using the last metabolic evaluation with ^18^Fluorodeoxyglucose positron emission tomography-computed tomography (^18^FDG-PET-CT) according to PERCIST criteria. Complete metabolic response (CMR) was defined by normalization of all lesions to at or below Standardized Uptake Value (SUV) of liver background_._ Partial metabolic response (PMR) was defined by a ≥50% decrease in the sum of all target lesion baseline SUV. Progressive metabolic disease (PMD) was defined by a ≥50% increase in the nadir sum of all target or new evaluable lesion SUV. Stable metabolic disease (SMD) was defined as condition that did not meet previous criteria.
