# Supplementary material for: Impact of BRAFV600E mutation on aggressiveness and outcomes in adult clonal histiocytosis
Source: Front Immunol. 2023 Sep 22;14:1260193. doi: 10.3389/fimmu.2023.1260193 (PMC10556468; doi:10.3389/fimmu.2023.1260193)
Supplement: Supplementary file 3 [file Table_2.docx]

**Supplementary table 2:** Logistic regression analysis for the factors associated with *BRAF^V600E^* mutation

|  | **Odds Ratio** | **95% CI for Odds Ratio** | **P value** | **Odds Ratio** | **95% CI for Odds Ratio** | **P value** |
| --- | --- | --- | --- | --- | --- | --- |
| Age>62 years | 2.50 | 0.5399-12.92 | 0.20505 |  |  |  |
| Men | 0.2045 | 0.03381-0.9981 | 0.0607 |  |  |  |
| **Type of histiocytosis** |  |  |  |  |  |  |
| ECD | 0.9524 | 0.1836-4.689 | 0.9521 |  |  |  |
| LCH | 2.0 | 0.4096-10.16 | 0.3899 |  |  |  |
| ECD/LCH | 3.556 | 0.3001-82.98 | 0.3268 |  |  |  |
| RDD | Not possible |  |  |  |  |  |
| **Clinical presentation** |  |  |  |  |  |  |
| Unicentric disease | 0.08889 | 0.004335-0.6210 | 0.0363* |  |  |  |
| Multicentric disease | 11.25 | 1.610-230.7 | 0.0363* | 3.226 | 0.1665-137.4 | 0.4572 |
| Risk organ involvement | 14.63 | 2.555-128 | 0.0056* | 4.566 | 0.2491-157.5 | 0.3179 |
| Bone involvement | 2.045 | 0.3453-16.75 | 0.4512 |  |  |  |
| Cardiovascular system | 5.833 | 0.9653-49.71 | 0.0686 |  |  |  |
| Lung | 1.037 | 0.1187-7.504 | 0.9712 |  |  |  |
| Endocrine system | 8.40 | 1.422-72.31 | 0.0280* |  |  |  |
| Digestive system | 2.500 | 0.4901-13.81 | 0.2734 |  |  |  |
| Retroperitoneal system | 1.833 | 0.3716-9.383 | 0.4548 |  |  |  |
| Naso-sinusal | 0.700 | 0.003-8.319 | 0.7826 |  |  |  |
| Eyes | 4.00 | 0.6237-34.41 | 0.1580 |  |  |  |
| Brain | 12.50 | 1.589-268 | 0.0349* | 4.566 | 0.2491-157.5 | 0.3838 |
| **Hematological condition** |  |  |  |  |  |  |
| Myeloid neoplasm | Not possible |  |  |  |  |  |
| Clonal hematopoiesis | 0.4500 | 0.05041-3.491 | 0.4499 |  |  |  |
| **Outcomes** |  |  |  |  |  |  |
| Partial metabolic response | 9.33 | 1.099-204.6 | 0.0671 | 0.05597 | 0.001019-0.9793 | 0.0814 |
| Stable metabolic disease | 4.0 | 0.7118-26.60 | 0.1250 |  |  |  |
| Complete metabolic response | Not possible |  |  |  |  |  |
| Progressive metabolic disease | 0.9167 | 0.1022-6.845 | 0.9323 |  |  |  |
| Death | 9.143 | 1.108-197.2 | 0.066 | 0.1290 | 0.01634-3.367 | 0.260 |
